# Mediating effect of inflammation on the association between glucocorticoid response-related polymorphism and metabolic syndrome in patients with schizophrenia: Evidence from the FondaMental Academic Centers of Expertise for Schizophrenia (FACE-SZ) cohort

**DOI:** 10.1016/j.bbih.2026.101391

**Published:** 2026-10-17

**Authors:** Kaori Saitoh, Mohamed Lajnef, Marion Leboyer, Myrtille Andre, Christelle Andrieu, Bruno Aouizerate, Maud Apavou, Caroline Barau, Jeremy Bernard, Jihène Bouassida, Delphine Capdevielle, Isabelle Chereau, Julie Clauss-Kobayashi, Jean-Michel Dorey, Caroline Dubertret, Eric Fakra, Guillaume Fond, Tudi Goze, Sylvain Leignier, Pierre-Michel Llorca, David Misdrahi, Nicolas Oriol, Baptiste Pignon, Romain Rey, Paul Roux, Jasmina Mallet, Franck Schürhoff, Benoit Schorr, Mathieu Urbach, Etienne Very, Ching-Lien Wu, the FACE-SZ (FondaMental Academic Centers of Expertise for Schizophrenia) collaborators, M. Andre, C. Andrieu-Haller, B. Aouizerate, F. Berna, O. Blanc, D. Capdevielle, M. Cléry, I. Chereau-Boudet, J. Clauss-Kobayashi, N. Coulon, R. Dassing, J.M. Dorey, C. Dubertret, T. D'Amato, A. Esselin, E. Fakra, G. Fond, F. Gabayet, T. Goze, M. Jarroir, D. Lacelle, M. Leboyer, S. Leignier, P.M. Llorca, J. Mallet, Z. Marongiu, E. Metairie, T. Michel, D. Misdrahi, N. Oriol, C. Passerieux, B. Pignon, P. Peri, C. Portalier, A. Couturier, P. Carrillo, R. Rey, C. Roman, P. Roux, B. Schorr, F. Schürhoff, A. Szöke, A. Tessier, M. Urbach, E. Very, A. Zinetti-Bertschy, Ophélia Godin, Wahid Boukouaci

**Affiliations:** a Univ Paris Est Créteil (UPEC), INSERM U955, IMRB, Translational Neuropsychiatry, Créteil, F-94010, France; b Fondation FondaMental, Créteil, 94000, France; c AP-HP, Hôpitaux Universitaires Henri Mondor, Département Médico-Universitaire de Psychiatrie et d’Addictologie (DMU IMPACT), Créteil, F-94010, France; d Service Universitaire de Psychiatrie Adulte, Hôpital la Colombière, CHRU Montpellier, Université Montpellier 1, Inserm, Montpellier, 1061, France; e AP-HM, Service Universitaire de Psychiatrie et d'addictologie , Hôpital Sainte Marguerite, Marseille, 13009, France; f University Department of General Psychiatry, Charles Perrens Hospital, Bordeaux, F-33076, France; g Laboratory of Nutrition and Integrative Neurobiology (UMR INRA 1286), University of Bordeaux, Bordeaux, France; h Sorbonne Université, Inserm, CNRS, Centre d’Immunologie et des Maladies Infectieuses (CIMI-Paris), Hôpital Pitié-Salpêtrière, Paris, France; i Division of Mental Health and Addiction, Centre for Precision Psychiatry, University of Oslo and Oslo University Hospital, Oslo, Norway; j Plateforme de Ressources Biologiques, AP-HP, Hôpitaux Universitaires Henri Mondor, Créteil, F94010, France; k Department of Pharmacology and Toxicology, Temerty Faculty of Medicine, University of Toronto, Toronto, ON, M5S 1A8, Canada; l University of Clermont Auvergne, CMP-B CHU, CNRS, Clermont Auvergne INP, Institut Pascal, Clermont-Ferrand, F-63000, France; m Hôpitaux Universitaires de Strasbourg, Université de Strasbourg, INSERM U1329, Fédération de Médecine Translationnelle de Strasbourg, Strasbourg, France; n INSERM U1028, CNRS UMR5292, Centre de Recherche en Neurosciences de Lyon, Université Claude Bernard Lyon 1, Equipe PSYR2, Centre Hospitalier Le Vinatier, Pole Est, 95 bd Pinel, BP 30039, 69678 Bron Cedex, France; o AP-HP, Groupe Hospitalo-universitaire AP-HP Nord, DMU ESPRIT, Service de Psychiatrie et Addictologie, Hôpital Louis Mourier, Colombes, France; p Université de Paris Inserm UMR1266, Sorbonne Paris Cité, Faculté de Médecine, France; q Department of Psychiatry, University Hospital of Saint-Etienne, Saint-Etienne, France; r Department of Psychiatry, Psychotherapies, Art-therapy, Toulouse University Hospital, Equipe de Recherche sur les Rationalités Philosophiques et les Savoirs - EA3051, Université de Toulouse - Jean Jaurès, Toulouse, France; s Centre Référent de Réhabilitation Psychosociale et de Remédiation Cognitive (C3R), CH Alpes Isère, Bordeaux, France; t University of Bordeaux, Aquitaine Institute for Cognitive and Integrative Neuroscience (CNRS UMR 5287-INCIA), Bordeaux, France; u Department of Adult Psychiatry, Charles Perrens Hospital, Bordeaux, France; v Centre Hospitalier de Versailles, Service Universitaire de Psychiatrie d’Adultes et d’Addictologie, Le Chesnay, 78150, France; w MOODS Team, INSERM 1018, CESP, Université de Versailles Saint-Quentin-En-Yvelines, Université Paris Saclay, Orsay, 91400, France; x Service Universitaire de Psychiatrie, EPSM Loiret, CHU Orléans, Orléans, France; y Equipe BCLINE, Laboratoire Li2rso, Université d'Orléans, Orléans, France

**Keywords:** Schizophrenia, Metabolic syndrome, GLCCI1, Glucocorticoid response, Inflammation

## Abstract

**Background:**

Dysregulation of the hypothalamic-pituitary-adrenal (HPA) axis and impaired glucocorticoid sensitivity may contribute to the inflammatory and metabolic abnormalities observed in schizophrenia (SZ), including metabolic syndrome (MetS), a major contributor to excess morbidity and premature mortality. However, the contribution of glucocorticoid response-related genetic variants to these abnormalities remains underexplored.

**Method:**

Two hundred and sixty-one patients with SZ were genotyped for two *NR3C1* polymorphisms (rs41423247, G > C and rs6198, T > C) and one *GLCCI1* polymorphism (rs37972, C > T). Logistic regression analyses were used to assess associations between genotypes and inflammation, evaluated by high-sensitivity C-reactive protein (hsCRP), and clinical features, including early-onset SZ (EOS), treatment-resistant SZ (TRS), suicidal attempts (SA) and MetS. Mediation analysis was used to evaluate the role of inflammation in genotype-MetS associations.

**Results:**

No significant association was observed between *NR3C1* polymorphisms and outcomes studied. In contrast, multivariate analysis showed that the *GLCCI1* rs37972 TT genotype was significantly associated with an increased likelihood of inflammation and MetS compared with TC/CC genotypes (inflammation: OR = 3.51, 95% CI 1.55-7.96, pFDR = 0.009; MetS: OR = 3.06, 95% CI 1.26-7.43, pFDR = 0.027). Additionally, mediation analysis revealed a significant indirect effect of the rs37972 TT genotype on MetS through inflammation, whereas the direct effect was not significant.

**Conclusion:**

The *GLCCI1* rs37972 polymorphism may be associated with altered glucocorticoid sensitivity, systemic inflammation, and MetS in SZ. These findings suggest a potential genetic-inflammatory pathway underlying the relationship between stress regulation and metabolic comorbidity in SZ. Given the cross-sectional design and the absence of a control group, the results should be considered exploratory and warrant replication in larger independent cohorts.

## Highlights

• Schizophrenia (SZ) patients show stress vulnerability with HPA axis dysregulation.
• The GLCCI1 gene regulates the HPA axis and modulates systemic inflammation.
• GLCCI1 polymorphisms are linked to higher inflammation and MetS risk in SZ.
• Mediation analyses suggest a genetic-inflammatory pathway in MetS.

## 1 Introduction

Schizophrenia (SZ) is a severe, complex and heterogeneous psychiatric disorder characterized by positive psychotic symptoms (i.e., delusions or hallucinations), as well as negative, disorganized and cognitive symptoms, in which neurobiological, immunological, and metabolic factors are intricately interconnected (Rahman and Lauriello, 2016). Although its precise aetiology remains elusive, it is now clearly considered as being a multisystem disorder given the elevated burden of medical comorbidities, including cardiovascular disorders, metabolic syndrome (MetS) and autoimmune disorders, the majority of them likely related to the often observed immune system dysfunction (Hennekens et al., 2005; Eaton et al., 2006; Ermakov et al., 2022; Godin et al., 2023; Leboyer and Tamouza, 2024). Concurrently, numerous studies have reported elevated inflammatory markers, including C-reactive protein (CRP) and interleukin (IL)-6 and IL-17, implicating chronic low-grade inflammation, which may be associated with cognitive impairment, treatment resistance, and increased metabolic burden in patients with SZ (Pape et al., 2019; Leboyer et al., 2021; Goldsmith et al., 2023). Altogether, these findings support the view that inflammatory processes may contribute both to clinical heterogeneity and to the excess somatic morbidity observed in SZ.

The hypothalamic-pituitary-adrenal (HPA) axis is a central neuroendocrine system that plays a critical role in coordinating stress response and regulating immune homeostasis (Herman et al., 2016). In physiological conditions, stress activates the release of corticotropin-releasing hormone (CRH) by the hypothalamus, which stimulates the anterior pituitary gland to secrete adrenocorticotropic hormone (ACTH). The latter then triggers the adrenal glands to release glucocorticoids, such as cortisol, into the blood from the adrenal cortex (Keller-Wood and Dallman, 1984; Herman et al., 2012). Cortisol exerts negative feedback on the HPA axis and contributes to the control of inflammatory activity (De Kloet et al., 1998; de Kloet et al., 2005). However, under chronic stress conditions, the HPA axis's negative feedback loop becomes impaired, leading to altered glucocorticoid sensitivity and dysregulated immune-inflammatory responses (Szczepankiewicz et al., 2011b).

Increasing evidence indicates that HPA axis dysregulation has been identified as an important interacting factor in the development and progression of SZ (Nikolić et al., 2024; Qi et al., 2024). Patients with SZ exhibit heightened vulnerability to stress and dysregulated cortisol secretion, frequently accompanied by severe clinical features, including early onset (EOS), treatment resistant (TRS), suicidal attempts (SA) and MetS (Wang, 2005; Mondelli et al., 2015; Broberg et al., 2018; Genis-Mendoza et al., 2022). Indeed, several studies have demonstrated an imbalance between immune and neuroendocrine systems in SZ, characterized by disrupted cortisol secretion and elevated CRP levels, suggesting resistance to glucocorticoids (Miller et al., 2005; Ushakov et al., 2019; Inova et al., 2025).

The *NR3C1* gene (nuclear receptor subfamily 3 group C member 1) encodes the glucocorticoid receptor (GR), a ligand-activated transcription factor that mediates most physiological effects of cortisol. Upon cortisol binding, GR translocates from the cytoplasm to the nucleus to regulate the transcription of target genes, including *GLCCI1* (Glucocorticoid-Induced 1), involved in the regulation of inflammatory response and immune cell apoptosis (Chapman et al., 1996; Tantisira et al., 2011; Brouwer et al., 2012), which acts as a true bridge between hormonal signaling and immune-metabolic responses. Consequently, genetic variation affecting either GR function or downstream glucocorticoid-responsive pathways may contribute to interindividual differences in susceptibility to inflammatory and metabolic abnormalities.

Two functional polymorphisms in the *NR3C1* gene were selected a priori because of their established biological relevance to glucocorticoid receptor function: *Bcl*I G > C (rs41423247) and exon 9β T > C (rs6198). In previous studies, these variants have been associated with altered GR sensitivity and increased production of the dominant-negative GRβ isoform, respectively (Derijk et al., 2001; Walker et al., 2008; Chan et al., 2010; Cuzzoni et al., 2012; Upthegrove and Khandaker, 2019; Castro-Vale et al., 2021; Palmer et al., 2024). Both polymorphisms have also been implicated in major depressive disorder and in the predominance of depressive symptoms in the course of bipolar disorder (Szczepankiewicz et al., 2011a; Peng et al., 2018). In parallel, the *GLCCI1* rs37972 (C > T) polymorphism, located in an intronic region, was selected because it has been associated with reduced glucocorticoid treatment response, notably in asthma, and with lower GLCCI1 expression in functional studies (Tantisira et al., 2011; Hu et al., 2021). Taken together, these data support the biological relevance of these three candidate polymorphisms for investigating the relationships among glucocorticoid signaling, inflammation, and metabolic abnormalities in SZ.

Given the growing evidence for HPA-axis dysregulation and immune-metabolic abnormalities in SZ, we investigated the potential impact of three glucocorticoid response-related polymorphisms (rs41423247 G > C and rs6198 T > C located in the *NR3C1* gene, and rs37972 C- > T located in the *GLCCI1* gene) on inflammatory regulation and major clinical outcomes, including EOS, SA risk, TRS and MetS.

## 2 Material and methods

### 2.1 Participants

The FondaMental Academic Centers of Expertise for Schizophrenia (FACE-SZ) cohort is drawn from an ongoing French national network of 10 schizophrenia expert centers (Bordeaux, Clermont-Ferrand, Colombes, Créteil, Grenoble, Lyon, Marseille, Montpellier, Strasbourg and Versailles), set up by the FondaMental Foundation (www.fondation-fondamental.org) (Schürhoff et al., 2015). This study included all 16-year-old outpatients with a DSM-IV-TR diagnosis of SZ or schizoaffective disorder who were clinically stable, as defined by the absence of hospitalization or treatment modifications during the 4 weeks before examination. After consent, patients were enrolled in the cohort, which underwent annual visits during which biological screening and standardized clinical evaluations were conducted to provide individualized treatment recommendations (Schürhoff et al., 2015). The study is carried out in agreement with ethical principles for medical research involving humans (WMA, Declaration of Helsinki). The relevant ethical review board (Comité de Protection des Personnes Ile de France IX; January 18, 2010) approved the protocol, in accordance with the French laws for non-interventional studies. All participants signed an informed consent and collected data were anonymized.

For the present analysis, a subgroup of 261 patients belonging to the FACE-SZ cohort was selected based on the availability of data on three glucocorticoid response-related polymorphisms, TRS, EOS, SA, MetS, and circulating high-sensitivity C-reactive protein (hsCRP) levels. A participant flow diagram describing the derivation of the analytical sample is provided in Supplementary Fig. 1.

Sociodemographic data, physical health exam, and clinical symptoms were systematically assessed. Body mass index (BMI) was calculated from measured weight and height. Current psychotic and depressive symptomatology were evaluated using the Positive And Negative Syndrome Scale (PANSS) (Kay SR et al., 1987) and the Calgary Depression Rating Scale for Schizophrenia (CDRS) (Addington D et al., 1993), respectively. Daily global functioning was evaluated with the Global Assessment of Functioning Scale (GAF) (Startup M et al., 2002). Current and past alcohol and cannabis use disorders were defined according to the SCID.

### 2.2 Outcomes

Five severe clinical and biological characteristics were defined as outcome variables: (i) Inflammatory status was assessed using previously measured circulating levels of hsCRP quantified with an electro-chemiluminescence-based assay (MesoScale Discovery (MSD) Human V-PLEX, Rockville, Maryland, USA), as reported in previous studies (Leboyer et al., 2021). Systemic inflammation was defined as hsCRP > 3 mg/L; (ii) MetS was defined according to the International Diabetes Federation criteria, requiring the presence of 3 or more of the following 5 components: high waist circumference (>94 cm for men and > 80 cm for women), hypertriglyceridemia (≥1.7 mmol/L or on lipid-lowering medication), low HDL cholesterol level (<1.03 mmol/L in men and < 1.29 mmol/L in women), high blood pressure (≥130/85 mmHg or on antihypertensive medication), and high fasting glucose concentration (≥5.6 mmol/L or on glucose-lowering medication). Patients were categorized as MetS+ (with MetS) or MetS− (without MetS); (iii) Early-Onset SZ (EOS) was defined as a diagnosis of schizophrenia with an age of onset before 18 years; (iv) Treatment-Resistant SZ (TRS) was defined as current treatment with clozapine (CLZ) (Leboyer M et al., 2021; Fond G et al., 2019); (v) Suicidal attempts (SA) were evaluated based on lifetime history of suicidal attempts reported by the patient.

### 2.3 Genotyping

Genomic DNA was extracted from EDTA-treated peripheral blood samples. Three single nucleotide polymorphisms (SNPs) herein studied, i.e., *GLCCl1* gene (rs37972 C- > T) and *NR3C1* gene (rs41423247 G- > C and rs6198 T- > C), were analyzed by a TaqMan® 5′-nuclease assay (Applied Biosystems®, Foster City, CA, USA) with allele-specific fluorogenic oligonucleotide probes. The following pre-developed TaqMan® assay genotyping kits were used: C____970132_20, C__86507873_10 and C___8951023_10.

### 2.4 Statistical analysis

Data are presented as median and interquartile range (IQR) for continuous variables, and number/percentage for categorical variables. Demographic and clinical characteristics were compared using the Kruskal-Wallis test for continuous variables and the chi-squared test for categorical variables. Deviation from Hardy-Weinberg equilibrium was tested using a chi-squared goodness-of-fit test.

Univariate associations between each polymorphism (GLCCI1 rs37972, NR3C1 rs41423247, and rs6198) and the study outcomes were assessed using chi-squared tests. For *GLCCI1* rs37972, additional logistic regression analyses were performed by comparing TT homozygotes with TC/CC carriers as the reference group. Odds ratios (ORs) and 95% confidence intervals (CIs) were calculated.

Multivariable logistic regression models were then performed to evaluate the association between the *GLCCI1* rs37972 TT genotype and the outcomes (EOS, MetS, inflammation), adjusting for age, sex, BMI, current smoking status, and CTQ-total and PANSS-total scores (Model 1), and additionally for EOS, MetS, or inflammation, depending on the outcome considered (Model 2). Missing data were handled using multiple imputation by chained equations (50 imputed datasets) under the assumption that data were missing at random.

Additive interaction between the *GLCCI1* rs37972 TT genotype and elevated CRP status in the occurrence of MetS was examined using Poisson regression within a generalized linear model (GLM) framework. Four groups were defined by TT genotype and CRP status [TT(−)CRP(−), TT(−)CRP(+), TT(+)CRP(−), TT(+)CRP(+)], with TT(−)CRP(−) groupe as the reference. Relative risks (RRs) and 95% CIs were estimated for each group. Additive interaction indices, including the relative excess risk due to interaction (RERI), the proportion attributable to interaction (PA), and the synergy index (S), were calculated following Knol et al. (2011) (see Supplementary Data).

To further explore the relationship between *GLCCI1* rs37972, inflammation, and MetS, a mediation analysis was used to test for a mediating effect of CRP status on the association between the *GLCCI1* rs37972 TT genotype and MetS, with direct, indirect, and total effects estimated via bootstrapping (5000 resamples). Both interaction and mediation models were adjusted for age, sex, current smoking status, antipsychotic treatment, and CTQ-total and PANSS-total scores. BMI was not included in these models because it is a component of the MetS definition.

Given the multiple statistical comparisons, secondary analyses, including interaction and mediation analyses, were considered exploratory. The primary univariable and multivariable logistic regression analyses evaluating the associations between the *GLCCI1* rs37972 TT genotype and the study outcomes were corrected for multiple testing using the Benjamini-Hochberg false discovery rate (FDR) procedure, with statistical significance defined by FDR-adjusted p-values (pFDR). Exploratory interaction and mediation analyses were not included in the FDR correction.

Sensitivity analyses were conducted by repeating the primary univariable and multivariable logistic regression analyses after excluding participants with hsCRP concentrations >10 mg/L, indicative of acute inflammation, using the same statistical models and covariate adjustments.

No formal a priori sample size calculation was performed, given the exploratory candidate-gene design of this study.

All analyses were performed using SPSS version 29 (IBM Corp., Armonk, NY, USA) and Jamovi version 2.3.28. All tests were two-sided, and p < 0.05 was considered statistically significant unless otherwise specified.

## 3 Results

### 3.1 Sample characteristics

A total of 261 patients with SZ were genotyped for one polymorphism in the *GLCCI1* gene (rs37972) and two polymorphisms in the *NR3C1* gene (rs41423247 and rs6198). For *GLCCI1* rs37972, genotype frequencies were 32 (12.3%) for TT, 122 (46.7%) for TC, and 107 (41.0%) for CC, corresponding to a minor allele frequency (MAF) of 0.36 for the T allele. For *NR3C1* rs41423247, genotype frequencies were 21 (8.0%) for CC, 123 (47.1%) for GC, and 117 (44.8%) for GG (MAF of the C allele = 0.32). For *NR3C1* rs6198, genotype frequencies were 6 (2.3%) for CC, 66 (25.3%) for TC, and 189 (72.4%) for TT (MAF of the C allele = 0.15). All genotype distributions were consistent with Hardy-Weinberg equilibrium (Table 1).

**Table 1:** Demographic, clinical, and treatment characteristics by GLCCI1 rs37972 genotype and genetic characteristics of the study cohort.

	Missing value (%)	All (N = 261)	CC (N = 107)	TC (N = 122)	TT (N = 32)	p-value
**Median (IQR) or Number (%)**						

**Age**	0	30 (25-37)	30 (25-38)	30 (25-36)	29 (23-35)	0.494
**Sex (male)#**	0	193 (73.9%)	77 (72.0%)	91 (74.6%)	25 (78.1%)	0.765
**BMI (kg/m^2^)**	9.2	26 (24-31)	26 (23-31)	27 (23-31)	27 (25-31)	0.946
**CTQ-Total**	0.4	39 (33-47)	37 (32-47)	41 (34-49)	40 (31-47)	0.193
**Current smokers#**	1.9	133 (52.0%)	51 (49.0%)	68 (55.7%)	14 (46.7%)	0.499
**CDRS**	0.4	2 (0-5)	2 (0-6)	2 (0-5)	4 (0-8)	0.549
**GAF**	1.1	50 (41-60)	49 (41-60)	51 (42-61)	50 (40-60)	0.307
**PANSS-Positive**	1.9	13 (9-17)	14 (10-18)	12 (9-17)	15 (10-18)	0.325
**PANSS-Negative**	1.5	16 (12-24)	18 (12-25)	15 (11-22)	18 (14-24)	0.078
**PANSS-General**	1.9	32 (25-40)	31 (23-44)	31 (25-37)	34 (30-42)	0.196
**PANSS-Total**	2.3	63 (47-77)	61 (46-88)	61 (46-73)	69 (59-82)	0.099
**Antipsychotics#**	4.6	238 (95.6%)	93 (93.0%)	115 (98.3%)	30 (93.8%)	0.145
**Antidepressants#**	4.6	79 (31.7%)	26 (26.0%)	39 (33.3%)	14 (43.8%)	0.150
**Anticholinergics#**	4.6	40 (16.1%)	15 (15.0%)	20 (17.1%)	5 (15.6%)	0.914
**Anxiolytics#**	4.6	71 (28.5%)	30 (30.0%)	29 (24.8%)	12 (37.5%)	0.337
**Hypnotics#**	4.6	23 (9.2%)	10 (10.0%)	10 (8.5%)	3 (9.4%)	0.934

**Genetic characteristics of the cohort**						

***GLCCI1 rs37972*. TT/TC/CC**	0	32 (12.3%)/122 (46.7%)/107 (41.0%)	–	–	–	–
**MAF (T allele)**		0.36	–	–	–	–

***NR3C1 rs41423247*. CC/GC/GG**	0	21 (8.0%)/123 (47.1%)/117 (44.8%)	–	–	–	–
**MAF (C allele)**		0.32	–	–	–	–

***NR3C1 rs6198*. CC/TC/TT**	0	6 (2.3%)/66 (25.3%)/189 (72.4%)	–	–	–	–
**MAF (C allele)**		0.15	–	–	–	–

BMI: Body mass index, GAF: Global Assessment of Functioning, CDRS: Calgary Depression Rating Scale, PANSS: Positive And Negative Syndrome Scale, CTQ: Childhood Trauma Questionnaire, IQR: interquartile range. MAF: minor allele frequency. Chi-square tests (#) and the Kruskal-Wallis test were used. All genotype distributions were consistent with Hardy-Weinberg equilibrium.

The demographic and clinical characteristics of patients according to *GLCCI1* rs37972 genotype are presented in Table 1. The median (IQR) age was 30 (25-37) years old and 74% of individuals were male. The median BMI was 26 (24-31) kg/m^2^, 52.0% were current smokers, 95.6% were receiving antipsychotic treatment, and 31.7% were treated with antidepressants and/or anxiolytics. Anticholinergics and hypnotics were prescribed in 16.1% and 9.2% of patients, respectively. No significant differences were observed across rs37972 genotype groups for age, sex, BMI, CTQ-total score, smoking status, depressive symptoms, global functioning, psychotic symptom severity, or psychotropic treatment.

### 3.2 Associations between *NR3C1* polymorphisms and demographic characteristics, clinical variables and outcomes

Associations between the two NR3C1 polymorphisms and demographic and clinical characteristics are presented in Supplementary Table 1. No significant association was observed between rs41423247 or rs6198 and any of the study outcomes, including SA, MetS, EOS, TRS, and inflammation (Supplementary Table 2). Accordingly, subsequent analyses focused on the *GLCCI1* rs37972 polymorphism.

### 3.3 Associations between *GLCCI1* rs37972 and study outcomes

The associations between *GLCCI1* rs37972 and the clinical and inflammatory outcomes are presented in Table 2. Among the 261 patients, 49 (18.8%) experienced SA, while 103 (39.5%) showed signs of inflammation. A total of 72 (27.6%) had MetS and 53 (20.3%) TRS. EOS was identified in 95 patients (36.4%).

**Table 2:** Association of the *GLCCI1* rs37972 polymorphism with clinical outcomes in patients with schizophrenia (N = 261).

rs37972	presence of outcome	Genotype			p-value^1^	pFDR (p-value^1^)	Major allele carrier	OR(95%CI)	p-value^2^	pFDR (p-value^2^)
***Outcome***	All N = 261	TT (N = 32)	TC (N = 122)	CC (N = 107)			TC + CC (N = 229)	TT vs. Ref (TC + CC)		
SA	49 (18.8%)	7 (21.9%)	22 (18.0%)	20 (18.7%)	0.884	0.884	42 (18.3%)	1.25 (0.51-3.07)	0.632	0.790
MetS	72 (27.6%)	14 (43.8%)	34 (27.9%)	24 (22.4%)	0.060	0.100	58 (25.3%)	2.29 (1.07-4.90)	0.032 *	**0.065**
EOS	95 (36.4%)	17 (53.1%)	34 (28.9%)	44 (41.1%)	0.013 *	0.030 *	78 (34.1%)	2.19 (1.04-4.63)	0.039 *	**0.065**
TRS	53 (20.3%)	6 (18.8%)	19 (15.6%)	28 (26.2%)	0.135	0.169	47 (20.5%)	0.89 (0.35-2.30)	0.815	0.815
inflammation+	103 (39.5%)	20 (62.5%)	41 (33.6%)	42 (39.3%)	0.012 *	0.030 *	83 (36.2%)	2.93 (1.37-6.30)	0.006 **	0.030 *

p-value^1^: comparison with each genotype (CC vs. TC vs. TT) using chi-square tests. p-value^2^: comparison between TT and TC/CC (as reference) using logistic regression analysis. SA: suicidal attempts. MetS: metabolic syndrome. EOS: early onset schizophrenia. TRS: treatment-resistant schizophrenia. Inflammation+: above 3 mg/L for hsCRP. OR: odds ratio. 95%CI: 95% confidence interval. FDR-adjusted P values (pFDR) were calculated using the Benjamini-Hochberg procedure. Statistical significance is indicated as P < 0.05 (*), P < 0.01 (**).

Genotype-based comparisons revealed significant associations with EOS (p = 0.013, pFDR = 0.030) and inflammation (p = 0.012, pFDR = 0.030), while the association with MetS showed a trend toward significance (p = 0.060, pFDR = 0.100). No significant association was observed for SA or TRS.

When TT homozygotes were compared with C-allele carriers (TC/CC), the TT genotype was associated with significantly higher odds of MetS (OR = 2.29, 95% CI 1.07-4.90, p = 0.032), EOS (OR = 2.19, 95% CI 1.04-4.63, p = 0.039), and inflammation (OR = 2.93, 95% CI 1.37-6.30, p = 0.006). After Benjamini-Hochberg correction, the association between *GLCCI1* rs37972 and inflammation remained significant (pFDR = 0.030), whereas associations with MetS and EOS were attenuated (both pFDR = 0.065).

Sensitivity analyses excluding participants with hsCRP concentrations >10 mg/L yielded comparable effect sizes and significance levels, indicating that acute inflammatory conditions did not drive the primary findings (Supplementary Table 3).

### 3.4 Multivariable associations between *GLCCI1* rs37972 and clinical outcomes

The multivariable logistic regression analyses are presented in Table 3. After adjusting for age, sex, BMI, current smoking status, CTQ-total score and PANSS-total score (Model 1), and following FDR correction for multiple testing, individuals carrying the rs37972 TT genotype were significantly more likely to exhibit inflammation (OR = 3.51, 95% CI 1.55-7.96, pFDR = 0.009) and MetS (OR = 3.06, 95% CI 1.26-7.43, pFDR = 0.027). The association with EOS was no longer statistically significant (OR = 2.07, 95% CI 0.89-4.82, p = 0.093).

**Table 3:** Logistic regression analyses of the association between *GLCCI1* rs37972 TT genotype and early-onset schizophrenia, inflammation, and metabolic syndrome in patients with schizophrenia (N = 261).

rs37972	Model 1				Model 2			
	OR	(95%CI)	P-value	pFDR	OR	(95%CI)	P-value	pFDR
**All N = 261**	**EOS** (N = 83)							
CC + TC (N = 229)	Ref				-			
TT (N = 32)	2.07	(0.89-4.82)	0.093		**-**	**-**	**-**	
	**Inflammation** (N = 103)				**Inflammation** (N = 103)			
CC + TC (N = 229)	Ref				Ref			
TT (N = 32)	**3.51**	**(1.55-7.96)**	**0.003**	0.009**	**3.46**	**(1.50-7.96)**	**0,004**	0.013*
	**MetS** (N = 72)				**MetS** (N = 72)			
CC + TC (N = 229)	Ref				Ref			
TT (N = 32)	**3.06**	**(1.26-7.43)**	**0.013**	0.027*	**3.06**	**(1.23-7.66)**	**0.017**	0.046*

Logistic regression analyses were performed. Missing data for BMI, CTQ total score, current smoking status, and PANSS total score (11% of participants) were handled using multiple imputation. OR: odds ratio. 95%CI: 95% confidence interval. Ref: reference category. EOS: early onset schizophrenia. MetS: metabolic syndrome. FDR-adjusted P values (pFDR) were calculated using the Benjamini-Hochberg procedure. Statistical significance is indicated as P < 0.05 (*), P < 0.01 (**).

Model 1: adjusted for age, sex, BMI, CTQ total, current smoking status and PANSS-Total score.

Model 2: additionally adjusted for EOS, inflammation, or MetS, depending on the outcome analyzed.

These associations remained stable after additional adjustment for EOS, MetS, or inflammation, depending on the outcome examined (Model 2). The rs37972 TT genotype remained independently associated with inflammation (OR = 3.46, 95% CI 1.50-7.96, pFDR = 0.013) and MetS (OR = 3.06 95% CI 1.23-7.66, pFDR = 0.046).

Sensitivity analyses excluding participants with hsCRP >10 mg/L produced consistent results, confirming the robustness of the primary associations (Supplementary Table 4).

### 3.5 Association between combined *GLCCI1* rs37972 genotype and CRP status and metabolic syndrome

The association between combined *GLCCI1* rs37972 genotype and CRP status with MetS, after adjusting for age, sex, antipsychotic treatment, current smoking status, CTQ-total score and PANSS-total score, is presented in Table 4. Using the TT(−)/CRP(−) group as the reference, the risk of MetS was significantly higher among patients with elevated CRP alone (TT(−)/CRP(+); RR = 1.95, 95% CI: 1.24–3.10, p = 0.004), among carriers of the TT genotype with normal CRP levels (TT(+)/CRP(−); RR = 2.52, 95% CI: 1.06–5.24, p = 0.022), and among patients presenting both the TT genotype and elevated CRP (TT(+)/CRP(+); RR = 2.14, 95% CI: 1.01–4.16, p = 0.035).

**Table 4:** Association of combined *GLCCI1* rs37972 genotype and CRP status with metabolic syndrome in patients with schizophrenia.

Outcome: MetS		(N = 261)	RR	95%CI	p-value
*GLCCI1*(TT)-CRP status	**TT(−)CRP(−)**	**146**	**Ref**		
	**TT(−)CRP(+)**	**83**	**1.95**	1.24-3.10	**0.004**
	**TT(+)CRP(−)**	**12**	**2.52**	1.06-5.24	**0.022**
	**TT(+)CRP(+)**	**20**	**2.14**	1.01-4.16	**0.035**

Relative risks (RRs) and 95% confidence intervals (CIs) were estimated using Poisson regression with robust variance within a generalized linear model (GLM) framework. The TT(−)/CRP(−) group served as the reference category. CRP positivity was defined as hsCRP >3 mg/L, and TT positivity corresponded to homozygosity for the rs37972 TT genotype. The model was adjusted for age, sex, antipsychotic treatment, current smoking status, CTQ-total score, and PANSS-total score. RR: Relative Risk. 95%CI: 95% confidence interval. Ref: reference category.

Measures of additive interaction did not support a synergistic effect between rs37972 TT genotype and CRP positivity on MetS risk. The combined effect of both exposures did not exceed the sum of their individual effects on the additive scale.

### 3.6 Mediation analysis

The mediation model is presented in Fig. 1. After adjusting for age, sex, antipsychotic treatment, current smoking status, CTQ-total score and PANSS-total score, the indirect effect of the *GLCCI1* rs37972 TT genotype on MetS through inflammation was statistically significant (a × b: β = 0.029, p = 0.047), whereas the direct effect was not (c: β = 0.106, p = 0.080). The total effect of the TT genotype on MetS remained significant (β = 0.135, p = 0.025). These findings suggest that inflammation may statistically account for part of the association between the rs37972 TT genotype and MetS. However, given the cross-sectional design, these results should be interpreted as evidence of statistical mediation rather than proof of a causal biological mechanism.

**Fig. 1:**
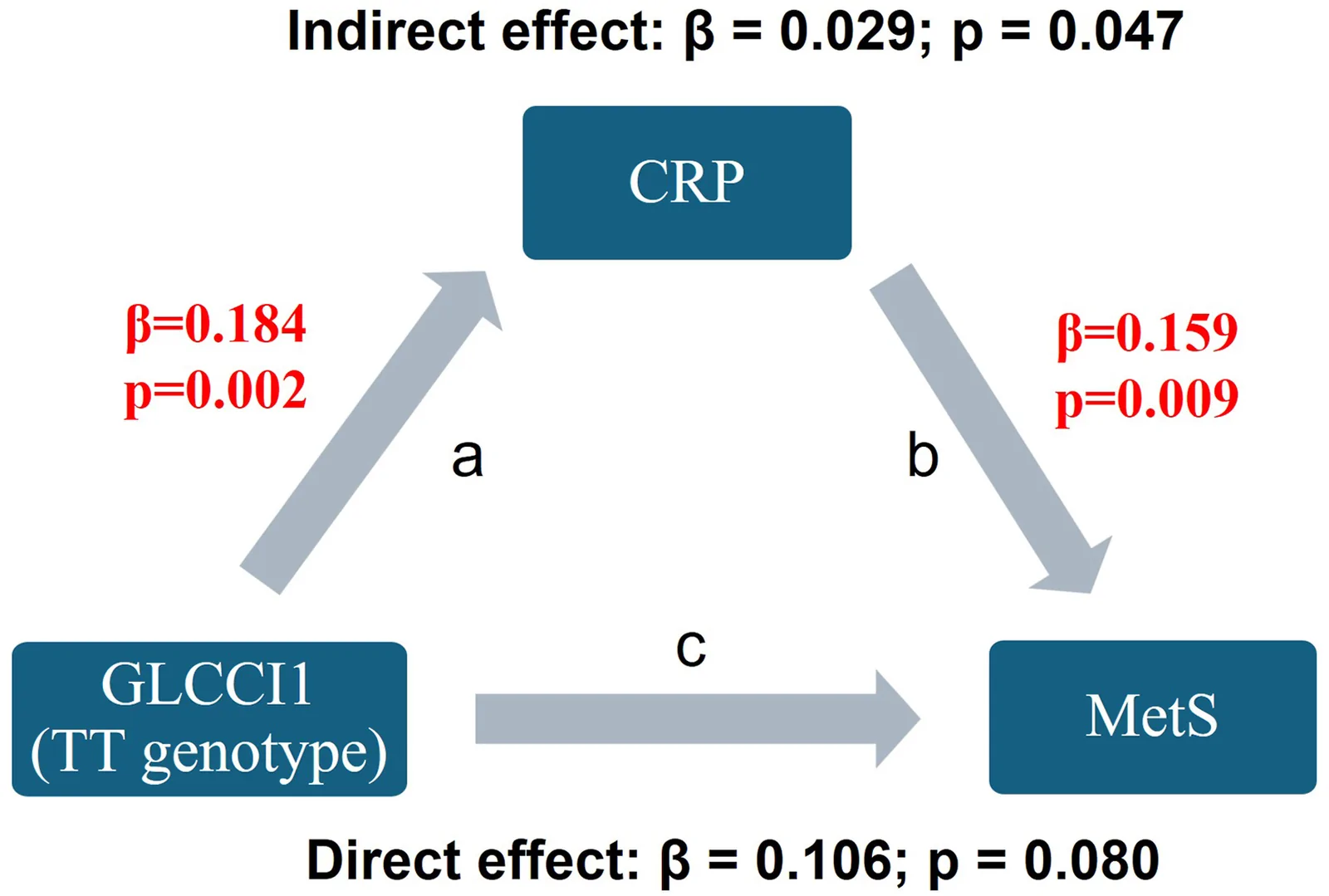
Mediation model: indirect effect of *GLCCI1* rs37972 polymorphism on MetS through CRP in patients with schizophrenia (N = 261). After adjusting for age, sex, antipsychotic treatment, current smoking status, CTQ-total score and PANSS-total score, the indirect pathway from the TT genotype of *GLCCI1* gene (rs37972) to MetS via inflammation (CRP) was significant (a×b: β = 0.029, p = 0.047). Whereas the direct pathway from the TT genotype at rs37972 to MetS was not (c: β = 0.106, p = 0.08). The total effect of the TT genotype at rs37972 on MetS remained significant (c + a×b: β = 0.135, p = 0.025). β: standardized regression coefficient; p: p-value.

## 4 Discussion

In this multicenter cohort of patients with SZ, we investigated whether common polymorphisms in genes involved in glucocorticoid signaling were associated with inflammatory and clinical outcomes. We showed, for the first time, that the *GLCCI1* rs37972 TT genotype, but not the two *NR3C1* polymorphisms examined, was independently associated with inflammation and MetS in patients with SZ. These associations remained significant after adjustment for relevant clinical covariates and sensitivity analyses. Although rs37972 was also associated with EOS in univariate analyses, this association was no longer significant after multivariable adjustment, suggesting that it may be explained by clinical confounding rather than an independent genetic effect. No associations were observed with TRS or SA.

The absence of significant association for *NR3C1* rs41423247 and rs6198 represents an important negative finding. These variants have previously been linked to altered glucocorticoid sensitivity and receptor isoform regulation in several inflammatory and metabolic disorders. However, our results do not support a major contribution of these polymorphisms to inflammatory or clinical heterogeneity in SZ. This discrepancy may reflect the complex genetic architecture of SZ, tissue-specific effects of *NR3C1* variants, gene-environment interactions, or the limited statistical power associated with relatively infrequent genotypes, particularly for rs6198 (CC genotype, n = 6). Consequently, subsequent analyses focused on *GLCCI1* rs37972, the only polymorphism showing significant associations in the primary analyses.

By contrast, the findings observed for *GLCCI1* rs37972 are biologically plausible. The minor allele frequency of this variant in our sample (0.36) was consistent with global reference frequencies (0.35 in TOPMed and 0.36 in gnomAD v4 genomes). This variant has been associated with impaired *GLCCI1* function, reduces sensitivity to glucocorticoids, leading to partial resistance to the anti-inflammatory effects of cortisol. Our results are consistent with previous studies showing that (i) the absence of *GLCCI1*, in animal models knockout (Glcci1−/−), is associated with an overexpression of pro-inflammatory cytokines and an exacerbation of the inflammatory response (Hu et al., 2018; Hu et al., 2021); (ii) the *GLCCI1* rs37972 minor allele is associated with a reduced response to corticosteroids, highlighting its potential impact on decreasing T cell apoptosis and maintaining prolonged immune activation (Tantisira et al., 2011; Brouwer et al., 2012; Zhang et al., 2024); (iii) chronic stress, sleep disorders, and certain metabolic pathologies are accompanied by a disruption of cortisol and an elevation of CRP levels (Laaksonen et al., 2004; Tolmay et al., 2012; Wright et al., 2015; Knight et al., 2021), reinforcing the systemic inflammatory profile.

In the context of SZ, impaired glucocorticoid signaling may contribute to persistent inflammation, reflected by elevated CRP levels (Fond et al., 2019), thereby increasing susceptibility to metabolic abnormalities and cardiovascular disease ( Ringen et al., 2014), supporting a role for GLCCI1 in the interplay between stress regulation, immune dysfunction, and metabolic burden.

Interestingly, mediation analyses suggested that inflammation may partially explain the association between the rs37972 TT genotype and MetS. The indirect effect through CRP remained statistically significant after adjustment for major confounders, whereas the direct effect was no longer significant. These findings are consistent with previous reports linking chronic low-grade inflammation to metabolic abnormalities in SZ, particularly among patients receiving antipsychotic treatment (Fawzi et al., 2011; Fond et al., 2018; Foiselle et al., 2022). Nevertheless, because of the cross-sectional design and the exploratory nature of this analysis, the mediation results should be interpreted as statistical rather than causal. Longitudinal studies will be required to determine whether impaired glucocorticoid responsiveness precedes the development of chronic inflammation and subsequent metabolic abnormalities.

Conversely, additive interaction analyses did not support a biological interaction between the rs37972 TT genotype and CRP positivity. Although each factor was individually associated with increased MetS risk, their combined effect did not exceed the sum of their individual effects, indicating the absence of additive synergy. This analysis was exploratory, and the limited number of TT homozygotes within each CRP stratum may have reduced the power to detect an additive interaction.

Several limitations should be acknowledged. First, the absence of a healthy control group precludes assessment of whether *GLCCI1* rs37972 influences susceptibility to SZ or whether genotype frequencies differ from those observed in the general population. Accordingly, our findings should be interpreted as reflecting inflammatory and metabolic heterogeneity within SZ rather than disease susceptibility. Second, although the overall sample size was comparable to that of previous candidate-gene studies in SZ, the relatively small number of rs37972 TT homozygotes (n = 32) reduced the statistical power to detect modest genetic effects and contributed to relatively wide confidence intervals for several estimates. Consequently, the absence of significant associations for treatment-resistant schizophrenia and suicidal attempts should be interpreted cautiously, as these analyses may have been underpowered. Replication in larger independent cohorts will be important to confirm the present findings. Third, although the primary analyses were adjusted for major clinical confounders, corrected for multiple testing, and confirmed in sensitivity analyses, residual confounding cannot be excluded. Fourth, genetic ancestry was not formally assessed, and self-reported ethnicity was not systematically collected in a format suitable for robust subgroup analyses. Population stratification therefore cannot be entirely excluded. In addition, only three candidate polymorphisms were investigated, and gene-environment interactions were not examined. Finally, inflammation was assessed using hsCRP alone, a nonspecific marker of systemic inflammation whose levels may be influenced by unrecognized inflammatory or infectious conditions.

Despite these limitations, this study has several strengths. It was conducted in a well-characterized multicenter cohort with detailed clinical, metabolic, and inflammatory phenotyping. Furthermore, the simultaneous evaluation of *GLCCI1* and *NR3C1* polymorphisms suggests that the observed associations were specific to *GLCCI1* rs37972 rather than a general feature of glucocorticoid signaling genes. The consistency of the findings across multivariable analyses, FDR correction, and sensitivity analyses further supports the robustness of the observed associations.

Overall, these findings identify *GLCCI1* as a promising candidate gene linking glucocorticoid responsiveness to immunometabolic dysfunction in SZ. Replication in larger, ethnically diverse cohorts and functional studies will be required to elucidate the molecular mechanisms underlying these associations. Longitudinal investigations incorporating repeated inflammatory measurements and broader genomic approaches should further clarify the temporal relationships between *GLCCI1* variation, chronic inflammation, and metabolic syndrome, and determine whether similar mechanisms contribute to other psychiatric disorders characterized by HPA-axis dysregulation, including bipolar disorder and major depressive disorder.

## 5 Conclusion

In conclusion, this study identifies the *GLCCI1* rs37972 polymorphism as a potential genetic determinant of inflammatory and metabolic vulnerability within SZ, rather than SZ susceptibility itself. The association of the rs37972 TT genotype with both low-grade inflammation and MetS, together with evidence of statistical mediation by inflammation, supports a role for altered glucocorticoid responsiveness in the immunometabolic dysregulation observed in SZ. Although these findings require replication in larger, independent cohorts and functional validation, they highlight *GLCCI1* as a promising candidate linking HPA-axis dysfunction, chronic inflammation, and metabolic complications in SZ, and may ultimately contribute to improved biological stratification and personalized management of cardiometabolic risk in this population.

## CRediT authorship contribution statement

**Kaori Saitoh:** Conceptualization, Formal analysis, Methodology, Writing – original draft. **Mohamed Lajnef:** Methodology, Software, Writing – review & editing. **Marion Leboyer:** Supervision, Writing – review & editing, Resources. **Myrtille Andre:** Resources, Writing – review & editing. **Christelle Andrieu:** Resources, Writing – review & editing. **Bruno Aouizerate:** Resources, Writing – review & editing. **Maud Apavou:** Writing – review & editing. **Caroline Barau:** Resources, Writing – review & editing. **Jeremy Bernard:** Writing – review & editing. **Jihène Bouassida:** Writing – review & editing. **Delphine Capdevielle:** Resources, Writing – review & editing. **Isabelle Chereau:** Resources, Writing – review & editing. **Julie Clauss-Kobayashi:** Resources, Writing – review & editing. **Jean-Michel Dorey:** Resources, Writing – review & editing. **Caroline Dubertret:** Resources, Writing – review & editing. **Eric Fakra:** Resources, Writing – review & editing. **Guillaume Fond:** Resources, Writing – review & editing. **Tudi Goze:** Resources, Writing – review & editing. **Sylvain Leignier:** Resources, Writing – review & editing. **Pierre-Michel Llorca:** Resources, Writing – review & editing. **David Misdrahi:** Resources, Writing – review & editing. **Nicolas Oriol:** Resources, Writing – review & editing. **Baptiste Pignon:** Resources, Writing – review & editing. **Romain Rey:** Resources, Writing – review & editing. **Paul Roux:** Resources, Writing – review & editing. **Jasmina Mallet:** Resources, Writing – review & editing. **Franck Schürhoff:** Resources, Writing – review & editing. **Benoit Schorr:** Resources, Writing – review & editing. **Mathieu Urbach:** Resources, Writing – review & editing. **Etienne Very:** Resources, Writing – review & editing. **Ching-Lien Wu:** Resources, Writing – review & editing. **A. Zinetti-Bertschy:** Resources, Writing – review & editing. **Ophélia Godin:** Data curation, Methodology, Supervision, Writing – review & editing. **Wahid Boukouaci:** Conceptualization, Formal analysis, Methodology, Resources, Supervision, Writing – original draft.

## Ethics declaration

Written informed consent to take part in the study and to publish the article has been obtained from all participants or their legal representatives. The privacy rights of participants have been observed.

This study included organ or tissue donors. This study includes human biological material and consent was obtained by donors, or their next of kin or legal representatives, for use in this study and for publication of the article. The samples used in this research were not sourced from executed prisoners or prisoners of conscience.

This study was performed in compliance with relevant laws, regulatory frameworks and guidelines where the research took place. This study was approved by the Collection biologique des Réseaux Nationaux des Centres Experts de la Fondation FondaMental: BIOFACE. (Approval No. ID-RCB: 2013-A01286-39)

## Statement

We acknowledge the use of ChatGPT (OpenAI, GPT-4) for assistance with language editing, improving readability, and annotating code. All outputs were reviewed, validated, and verified entirely by the authors, who take full responsibility for the content of this manuscript.

## Financial support

This research was supported by the Fondation FondaMental, Institut National de la Santé et de la Recherche Médicale (INSERM), and by the Investissements d’Avenir program managed by the Agence National de la Recherche (ANR) under reference ANR-11-IDEX-0004-02 and ANR-10-COHO-10-01. This funding source had no role in the study design, data collection, analysis, preparation of the manuscript, or decision to submit the manuscript for publication.

## Declaration of competing interest

The authors declare that they have no known competing financial interests or personal relationships that could have appeared to influence the work reported in this paper.
