# Supplementary material for: Mediating effect of inflammation on the association between glucocorticoid response-related polymorphism and metabolic syndrome in patients with schizophrenia: Evidence from the FondaMental Academic Centers of Expertise for Schizophrenia (FACE-SZ) cohort
Source: Brain Behav Immun Health. 2026 Oct 17;58:101391. doi: 10.1016/j.bbih.2026.101391 (PMC13737410; doi:10.1016/j.bbih.2026.101391)
Supplement: Supplementary file 1 [file mmc1.docx]

**Supplementary table 1. Demographic and clinical characteristics of patients with schizophrenia according to *NR3C1* polymorphisms.**

| **All N=261** |  | **Missing value** | **All (N=261)** | **GG (N=117)** | **CG (N=123)** | **CC (N=21)** | **p-value** |
| --- | --- | --- | --- | --- | --- | --- | --- |
| **rs41423247** |  |  |  | **median (IQR) or Number (%)** | |  |  |
| **Age** |  | 0 | 30.0 (24.5-36.5) | 32.0 (25.0-38.0) | 29.0 (24.0-36.0) | 27.0 (24.5-32.5) | 0.111 |
| **Sex (male)#** |  | 0 | 193 (73.9%) | 76 (65.0%) | 98 (79.%) | 19 (90.5%) | 0.007 |
| **BMI (Kg/m2)** | | 9,2 | 26.4 (23.3-30.8) | 27.2 (23.5-30.9) | 26.1 (23.0-30.3) | 26.6 (24.4-29.3) | 0.607 |
| **CTQ-Total** |  | 0,4 | 39.0 (33.0-47.0) | 39.0 (33.0-49.0) | 39.5 (33.0-46.0) | 38.0 (31.5-49.0) | 0.936 |
| **Current smokers#** | | 1,9 | 133 (52.0%) | 56 (48.7%) | 67 (55.8%) | 10 (47.6%) | 0.504 |
| **Substance abuse#** | | 11.9 | 117 (50.9%) | 47 (47.5%) | 59 (53.6%) | 11 (52.4%) | 0.666 |
| **CDRS** |  | 0,4 | 2.0 (0.0-5.0) | 2.0 (0.0-5.0) | 2.0 (0.0-6.0) | 0.0 (0.0-3.0) | 0.073 |
| **GAF** |  | 1,1 | 50.0 (41.0-60.0) | 49.5 (40.0-60.0) | 51.0 (41.0-60.0) | 55.0 (41.0-65.0) | 0.419 |
| **PANSS-Positive** | | 1,9 | 13.0 (9.3-17.7) | 14.0 (10.0-18.0) | 13.0 (10.0-17.0) | 10.0 (8.0-15.0) | 0.057 |
| **PANSS-Negative** | | 1,5 | 16.0 (12.0-23.5) | 18.0 (12.0-24.0) | 16.0 (11.0-23.3) | 14.0 (8.0-20.0) | 0.437 |
| **PANSS-General** | | 1,9 | 32.0 (25.0-39.8) | 33.0 (27.3-44.0) | 31.0 (23.5-37.0) | 28.0 (23.0-37.0) | 0.037 |
| **PANSS-Total** | | 2,3 | 63.0 (47.0-77.0) | 64.0 (50.0-84.0) | 60.0 (47.5-74.0) | 56.0 (43.0-71.0) | 0.071 |
| **Antipsychotics#** | | 4.6 | 238 (95.6%) | 104 (93.7%) | 113 (96.6%) | 21 (100.0%) | 0.335 |
| **Antidepressants#** | | 4.6 | 79 (31.7%) | 35 (31.5%) | 38 (32.5%) | 6 (28.6%) | 0.938 |
| **Anticholinergics#** | | 4,6 | 40 (16.1%) | 16 (14.4%) | 22 (18.8%) | 2 (9.5%) | 0.463 |
| **Anxiolytics#** |  | 4,6 | 71 (28.5%) | 38 (34.2%) | 30 (25.6%) | 3 (14.3%) | 0.114 |
| **Hypnotics#** |  | 4,6 | 23 (9.2%) | 14 (12.6%) | 9 (7.7%) | 0 (0.0%) | 0.137 |
| **All N=261** |  | **Missing value** | **All (N=261)** | **TT (N=189)** | **CT (N=66)** | **CC (N=6)** | **p-value** |
| **rs6198** |  |  |  | **median (IQR) or Number (%)** | |  |  |
| **Age** |  | 0 | 30.0 (24.5-36.5) | 30.0 (24.4-37.0) | 30.0 (25.8-36.3) | 31.5 (27.3-39.8) | 0.751 |
| **Sex (male)#** |  | 0 | 193 (73.9%) | 138 (73.0%) | 52 (78.8%) | 3 (50.0%) | 0.263 |
| **BMI (Kg/m2)** | | 9,2 | 26.4 (23.3-30.8) | 26.3 (23.3-30.4) | 27.5 (23.2-31.0) | 24.4 (21.8-28.3) | 0.412 |
| **CTQ-Total** |  | 0,4 | 39.0 (33.0-47.0) | 39,5 (33.0-47.0) | 39.0 (33.0-46.3) | 40.0 (29.8-48.3) | 0.948 |
| **Current smokers#** | | 1,9 | 133 (52.0%) | 96 (51.9%) | 32 (49.2%) | 5 (83.3%) | 0.278 |
| **Substance abuse#** | | 11.9 | 117 (50.9%) | 85 (49.7) | 29 (53.7%) | 3 (60.0%) | 0.805 |
| **CDRS** |  | 0,4 | 2.0 (0.0-5.0) | 2.0 (0.0-6.0) | 2.0 (0.0-4.5) | 1.0 (0.0-7.0) | 0.784 |
| **GAF** |  | 1,1 | 50.0 (41.0-60.0) | 51.0 (41.0-61.0) | 49.0 (41.0-55.0) | 38.0 (24.0-56.3) | 0.119 |
| **PANSS-Positive** | | 1,9 | 13.0 (9.3-17.7) | 13.0 (10.0-17.0) | 13.0 (9.0-18.0) | 18.5 (11.3-27.0) | 0.216 |
| **PANSS-Negative** | | 1,5 | 16.0 (12.0-23.5) | 16.0 (11.0-22.0) | 19.0 (12.0-25.0) | 23.5 (11.8-26.3) | 0.151 |
| **PANSS-General** | | 1,9 | 32.0 (25.0-39.8) | 31.0 (24.0-38.0) | 33.5 (26.3-42.8) | 45.5 (26.3-52.0) | 0.083 |
| **PANSS-Total** | | 2,3 | 63.0 (47.0-77.0) | 60.0(46.5-75.0) | 66.0 (51.3-83.8) | 88.0 (49.5-105.5) | 0.065 |
| **Antipsychotics#** | | 4.6 | 238 (95.6%) | 172 (96.6%) | 61 (92.4%) | 5 (100.0%) | 0.324 |
| **Antidepressants#** | | 4.6 | 79 (31.7%) | 55 (30.9%) | 23 (34.8%) | 1 (20.0%) | 0.715 |
| **Anticholinergics#** | | 4,6 | 40 (16.1%) | 27 (15.2%) | 12 (18.2) | 1 (20.0%) | 0.826 |
| **Anxiolytics#** |  | 4,6 | 71 (28.5%) | 50 (28.1%) | 19 (28.8%) | 2 (40.0%) | 0.843 |
| **Hypnotics#** |  | 4,6 | 23 (9.2%) | 15 (8.4%) | 6 (9.1%) | 2 (40.0%) | 0.055 |
| BMI: Body mass index, GAF: Global Assessment of Functioning, CDRS: Calgary Depression Rating Scale, PANSS: Positive And Negative Syndrome Scale, CTQ: Childhood Trauma Questionnaire, MetS: metabolic syndrome, IQR: interquartile range. Chi-square tests (#) and the Kruskal-Wallis test were used. | | | | | | | |

**Supplementary Table 2. Associations between the *NR3C1* polymorphisms and outcomes in patients with schizophrenia (N=261)**

| **rs41423247** | presence of |  | Genotype | | | p-value^1^ |
| --- | --- | --- | --- | --- | --- | --- |
|  | outcome |  |  |  |  |  |
| ***Outcome*** | All N=261 |  | CC(N=21) | CG (N=123) | GG (N=117) |  |
| SA | 49 (18.8%) |  | 1 (4.8%) | 28 (22.8%) | 20 (17.1%) | 0.122 |
| MetS | 72 (27.6%) |  | 5 (23.8%) | 30 (24.4%) | 37 (31.6%) | 0.420 |
| EOS | 95 (36.4%) |  | 5 (23.8%) | 47 (38.2%) | 43 (36.8%) | 0.445 |
| TRS | 53 (20.3%) |  | 4 (19.0%) | 26 (21.1%) | 23 (19.7%) | 0.950 |
| inflammation+ | 103 (39.5%) |  | 10 (47.6%) | 47 (38.2%) | 46 (39.3%) | 0.717 |
| **rs6198** | presence of |  | Genotype | | | p-value^1^ |
|  | outcome |  |  |  |  |  |
| ***Outcome*** | All N=261 |  | CC (N=6) | CT (N=66) | TT (N=189) |  |
| SA | 49 (18.8%) |  | 1 (16.7%) | 12 (18.2%) | 36 (19.0%) | 0.979 |
| MetS | 72 (27.6%) |  | 1 (16.7%) | 18 (27.3%) | 53 (28.0%) | 0.827 |
| EOS | 95 (36.4%) |  | 2 (33.3%) | 24 (36.4%) | 69 (36.5%) | 0.987 |
| TRS | 53 (20.3%) |  | 1 (16.7%) | 14 (21.2%) | 38 (20.1%) | 0.957 |
| inflammation+ | 103 (39.5%) |  | 3 (50.0%) | 21 (31.8%) | 79 (41.8%) | 0.313 |
| p-value^1^: comparison with each genotype (CC vs. TC vs. TT) using chi-square tests. p-value^2^: comparison between TT and TC/CC (as reference) using logistic regression analysis. SA: suicidal attempts. MetS : metabolic syndrome. EOS: early onset schizophrenia. TRS: treatment-resistant schizophrenia. Inflammation+: above 3 mg/L for hsCRP. OR: odds ratio. 95%CI: 95% confidence interval. | | | | | | |

**Supplementary Table 3: Association of the *GLCCI1* rs37972 polymorphism with clinical outcomes in patients with schizophrenia after excluding participants with elevated CRP levels (>10 mg/L) (N = 234)**

| **rs37972** | **presence of  outcome** |  | **Genotype** | | | **p-value^1^** | **Major allele  carrier** | **OR(95%CI)** | **p-value^2^** |  |  |
| --- | --- | --- | --- | --- | --- | --- | --- | --- | --- | --- | --- |
| ***Outcome*** | All N=234 |  | TT (N=27) | TC (N=110) | CC (N=97) |  | TC+CC (N=207) | TT vs. Ref (TC+CC) |  |  |  |
| SA | 42 (17.9%) |  | 6 (22.2%) | 18 (16.4%) | 18 (18.6%) | 0.762 | 36 (17.4%) | 1.3 (0.51-3.60) | 0.538 |  |  |
| MetS | 59 (25.2%) |  | 12 (44.4%) | 26 (23.6%) | 21 (21.6%) | **0.048*** | 47 (22.7%) | 2.7 (1.19-6.22) | **0.014 *** |  |  |
| EOS | 83 (35.5%) |  | 14 (51.8%) | 31 (28.2%) | 38 (39.2%) | **0.043 *** | 69 (33.3%) | 2.0 (1.14-4.93) | 0.058 |  |  |
| TRS | 45 (19.2%) |  | 6 (22.2%) | 15 (13.6%) | 24 (24.7%) | 0.130 | 39 (19.2%) | 1.2 (0.45-3.25) | 0.675 |  |  |
| inflammation+ | 76 (32.5%) |  | 15 (55.6%) | 29 (26.4%) | 32 (33.0%) | **0.015 *** | 61 (29.5%) | 3.0 (1.32-6.76) | **0.006 **** |  |  |

p-value^1^: comparison with each genotype (CC vs. TC vs. TT) using chi-square tests. p-value^2^: comparison between TT and TC/CC (as reference) using logistic regression analysis. SA: suicidal attempts. MetS: metabolic syndrome. EOS: early onset schizophrenia. TRS: treatment-resistant schizophrenia. Inflammation+: above 3 mg/L for hsCRP. OR: odds ratio. 95%CI: 95% confidence interval. Statistical significance is indicated as P < 0.05 (*), P < 0.01 (**).

**Supplementary Table 4: Logistic regression analyses of the association between *GLCCI1* rs37972 TT genotype and early-onset schizophrenia, inflammation, and metabolic syndrome after excluding participants with elevated CRP levels (>10 mg/L) (N = 234)**

|  | Model 1 | | |  | Model 2 | | |  |
| --- | --- | --- | --- | --- | --- | --- | --- | --- |
| rs37972 | OR | (95%CI) | P-value |  | OR | (95%CI) | P-value |  |
| All N=234 | EOS (N=83) | | |  |  |  |  |  |
| CC+TC (N=207) | Ref |  |  |  | - |  |  |  |
| TT (N=27) | 1.99 | (0.81-4.92) | 0.135 |  | **-** | **-** | **-** |  |
|  | Inflammation (N=76) | | |  | Inflammation (N=76) | | |  |
| CC+TC (N=207) | Ref |  |  |  | Ref |  |  |  |
| TT (N=27) | **3.47** | **(1.48-8.15)** | **0.004** | ** | **3.41** | **(1.42-8.16)** | **0.006** | ** |
|  | MetS (N=59) | | |  | MetS (N=59) | | |  |
| CC+TC (N=207) | Ref |  |  |  | Ref |  |  |  |
| TT (N=27) | **3.64** | **(1.41-9.38)** | **0.008** | * | **3.63** | **(1.37-9.62)** | **0.010** | * |

Logistic regression analyses were performed. Missing data for BMI, CTQ total score, current smoking status, and PANSS total score (11% of participants) were handled using multiple imputation.

OR: odds ratio. 95%CI: 95% confidence interval. Ref: reference category. EOS: early onset schizophrenia. MetS: metabolic syndrome.

Model 1: adjusted for age, sex, BMI, CTQ total, current smoking status and PANSS-Total score.

Model 2: additionally adjusted for EOS, inflammation, or MetS, depending on the outcome analyzed.

**Supplementary Figure 1: Participant flow diagram**

SZ: schizophrenia; GR: Glucocorticoid response; SA: suicidal attempts; MetS: metabolic syndrome; EOS: early-onset schizophrenia; TRS: treatment-resistant schizophrenia; inflammation data were defined by CRP >3 mg/L.

**Supplementary data**

**Calculation of Measures of Additive Interaction**

To assess additive interaction between *GLCCI1* genotype and CRP status on the risk of metabolic syndrome. we calculated the Relative Excess Risk due to Interaction (RERI). the Attributable Proportion due to interaction (AP). and the Synergy Index (S). These indices are widely used to evaluate whether the joint effect of two risk factors exceeds the sum of their individual effects.

1. **Formulas**

RR11 = relative risk for double exposure TT(+)CRP(+)
RR10 = relative risk for TT(+)CRP(-)
RR01 = relative risk for TT(-)CRP(+)
RR00 = relative risk for reference group TT(-)CRP(-). set to 1.

**Relative Excess Risk due to Interaction (RERI):**

RERI = RR11 − RR10 − RR01 + 1

**Attributable Proportion due to interaction (AP):**

AP = RERI / RR11

**Synergy Index (S):**

S = (RR11 − 1) / [(RR10 − 1) + (RR01 − 1)]

2. **Interpretation**

**RERI > 0** indicates positive interaction (synergy) on the additive scale. meaning the combined effect is greater than the sum of individual effects.

**RERI = 0** or **RERI < 0** indicates no interaction or negative interaction.

**AP** reflects the proportion of risk among doubly exposed individuals TT(+)CRP(+) that is attributable to the interaction.

**S > 1** indicates synergy. **S = 1** or **S < 1** no synergy. between the *GLCCI1* genotype and CRP for the risk of metabolic syndrome.

**RERI < 0. AP < 0. and S < 1** indicate an antagonistic interaction on the additive scale (the combined exposure shows a lower effect than the sum of the individual effects). i.e.. no synergy between the *GLCCI1* genotype and CRP for the risk of metabolic syndrome.
